# Cassava mosaic disease: a review of a threat to cassava production in Zambia

**DOI:** 10.1007/s42161-019-00255-0

**Published:** 2019-02-12

**Authors:** Patrick Chiza Chikoti, Rabson Mpundu Mulenga, Mathias Tembo, Peter Sseruwagi

**Affiliations:** 1 Zambia Agriculture Research Institute, Mt. Makulu Central Research Station, P/B 7, Chilanga, Zambia; 2 Mikocheni Agricultural Research Institute, P.O. Box 6226, Dar es Salaam, Tanzania

**Keywords:** Southern Africa, Begomoviruses, Diagnostics, Epidemiology, Phytosanitation, Whitefly

## Abstract

Cassava (*Manihot esculenta* Crantz) is one of the most important root staple crops in Zambia. An estimated 30% of Zambians, over 4 million people, consume cassava as part of their daily diet. Cassava is mostly grown by subsistence farmers on fields of less than 1 ha. Cultivation of cassava is hampered by several biotic constraints, of which cassava mosaic disease (CMD) is currently the most important factor limiting cassava production in Zambia. CMD occurs in all the cassava-growing provinces and accounts for 50% to 70% of yield losses countrywide. Strategies to counter CMD were initiated in the early 1990s and included the release of CMD-resistant cassava cultivars. However, efforts to control CMD are limited because few growers plant these cultivars. More recently, to address the CMD problem, regular disease monitoring and diagnostic capabilities have been strengthened, and there is increased support for screening breeders materials. CMD is a rising threat to cassava production in Zambia. This review of CMD research on disease surveillance, CMD spread, yield losses, awareness campaigns and control options in Zambia over the past 25 years informs future control efforts and management strategies.

## Introduction

Cassava (*Manihot esculenta* Crantz) is the second most economically important crop in Zambia, after maize. The crop is widely grown in Northern, Luapula, North-Western and Western provinces, and in parts of Lusaka and Central provinces. Northern, Luapula and North-Western provinces are the major cassava-producing areas. An estimated 30% of the population, mostly subsistence farmers, depend on cassava as a staple crop and as a source of income. The cassava roots are a source of carbohydrates, with an estimated 25.3–35.7 g carbohydrate per 100 g dry weight (DW) (Bradbury and Holloway 1988). The cassava leaves are also consumed as a vegetable and are a source of vitamins, fibre, minerals and proteins. Proteins compose about 28.1 g/100 g (DW), comparable to the amounts reported for sweet potato (30.6 g/100 g DW) and peanut (26.6 g/100 g DW) (Wobeto et al. 2006). In Zambia, cassava accounts for 15% of national calorie consumption (Dorosh et al. 2007). Apart from traditional culinary uses, in urban towns such as Lusaka, Kitwe and Ndola, cassava is increasingly being used as a raw material in the production of starch, ethanol, beer and feed for livestock (Haggblade and Nyembe 2008). Although most of the crop is still consumed, in 2015, 22.3% of Zambian households sold cassava from their own farms (RALS 2016).

Cassava is believed to have been introduced to Zambia via the Congo basin where crops were well established by 1650 (Jones 1959). With the immigration of the Bemba people from the Lunda Luba Kingdom (now Democratic Republic of the Congo), cassava became an important crop in Northern Zambia by the 1700s. The importance of the crop has steadily increased in other parts of the country, including Copperbelt, Central and Eastern provinces where maize has been the traditional crop. The resilience of the cassava plant enables it to grow well under a wide range of agroecological zones, including zones where maize and other crops cannot thrive. Cassava is also a more attractive crop in these areas because it produces higher yields per unit of land than maize.

Cassava is cultivated as a tuberous root crop and propagated using stem cuttings. Stem cuttings can be entry points for diseases caused by pathogens, particularly viruses. One of the most important diseases of cassava is cassava mosaic disease (CMD), which is transmitted primarily by the vector whitefly (*Bemisia tabaci*) (Chant 1958). CMD is the most important threat to cassava production in Zambia. The disease is prevalent in most cassava fields (Chikoti et al. 2013a) and contributes to significant losses in yields (Muimba–Kankolongo et al. 1997). Numerous viruses cause CMD and they can occur as single or mixed infections. These viruses are well adapted to a range of environmental conditions and can adapt to plant resistance.

This first review of CMD in Zambia analyses the past and present research activities. We address the following topics: cassava production, CMD, epidemiology, causal organisms, distribution of cassava mosaic begomoviruses, and CMD management strategies that have been implemented or could be adopted in Zambia. Finally, we propose areas for future management.

## Cassava production

In Zambia, cassava is produced mainly by small-scale farmers. The majority of cassava producers are in Northern (210,706), Luapula (157,885), and North-Western (74,618) provinces, accounting for about 78.8% of all producers in Zambia (Sitko et al. 2011). Cassava cultivation has tripled in recent years, from 360,000 t in 1985 to 1,114,000 t in 2013. The increase in cassava cultivation is largely attributed to the withdrawal of maize subsides. Since 2005, the country has produced more than 1 million tonnes of cassava annually (FAOSTAT 2017). Cassava yields vary between producers using traditional cassava cultivars (1 t/ha) and improved cultivars (2–3.5 t/ha) (Sitko et al. 2011). Although cassava has the potential to yield as high as 20–40 t/ha (Plucknett et al. 2000), yields in Zambia average 5.8 t/ha (FAOSTAT 2017) compared with the African average of 8.4 t/ha (FAOSTAT 2017).

## Cassava mosaic disease

CMD is caused by viruses belonging to the genus *Begomovirus* in the family *Geminiviridae *(Hong et al. 1993). It is the single most important viral disease of cassava in Zambia and it also occurs in many neighbouring countries, including Tanzania, Malawi, Democratic Republic of the Congo, Zimbabwe, Mozambique and Angola.

## Symptoms

**Fig. 1:**
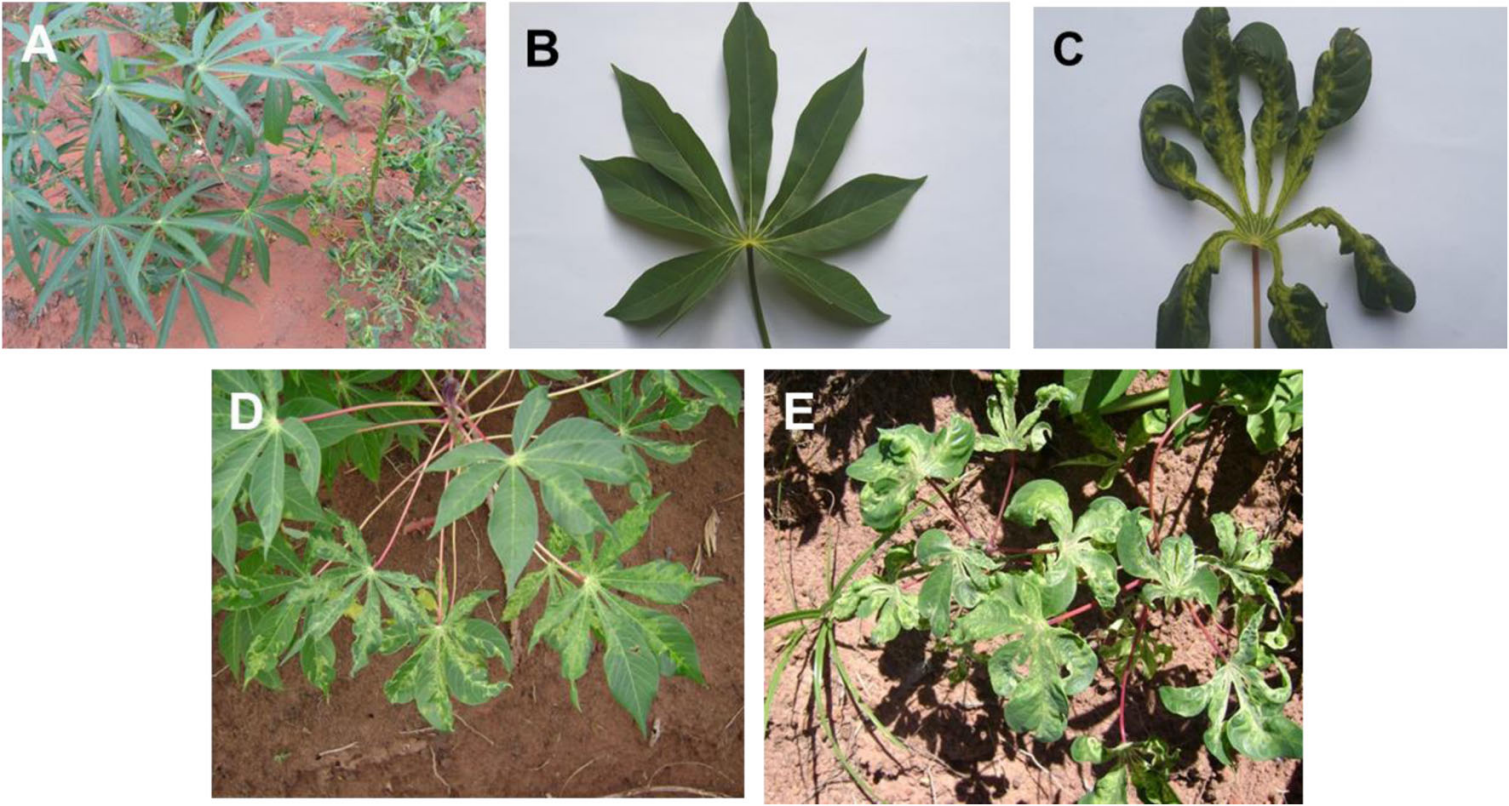
Symptoms of cassava mosaic disease (CMD). **a** A healthy cassava plant (left) and a plant infected with CMD (right); plants are same cultivar, Manyopola. **b** A healthy cassava leaf. **c** A cassava leaf showing severe CMD symptoms including leaf curling and chlorosis. **d** A cassava plant (cultivar Katobamputa) with a single infection of African cassava mosaic virus (ACMV) and **e** a plant of the same cultivar with a dual infection of ACMV and East African cassava mosaic virus (EACMV)

The symptoms of CMD are well characterised. Symptoms include distortion of leaf lamina, chlorotic mosaics, mottling and an overall reduction in plant size (Fig. 1a) compared with healthy leaves (Fig. 1b). Among the noticeable symptoms usually present in the field is a mosaic pattern on the leaves, the colouring of which can range from pale green to whitish yellow. The extent of chlorosis on the leaf surface varies between <5% to almost 100%. Another common feature observed in cassava fields is the extreme narrowing of the leaf near the base of the leaflets (Fig. 1c). However, symptoms can vary by both season and cultivar. Farmers generally grow a number of cultivars, both landraces and improved cultivars bred by Zambia Agriculture Research Institute (ZARI). Because these cultivars display varying CMD symptoms, CMD may go unnoticed despite the presence of symptoms. Indeed, studies conducted in 2009 showed that most farmers in Luapula Province were unable to recognise the symptoms of CMD (Chikoti 2011). Symptom expression in plants is influenced by the virus species infecting cassava plants and environmental factors. Work by Fondong et al. (2000) and Pita et al. (2001a) showed that symptoms can be exacerbated in plants that have mixed virus infection. Studies in Zambia have indicated severe symptoms in plants infected with African cassava mosaic virus (ACMV) and East African cassava mosaic virus (EACMV) (Chikoti et al. 2013a).

Expression of disease foliar symptoms is also influenced by soil fertility and water availability. Information on the abiotic factors that influence symptom expression in CMD are lacking in Zambia, however research on viral diseases in other regions has shown that low temperatures are associated with the development of severe symptoms (Gerik et al. 1990), whereas high temperatures are associated with attenuated symptoms. To test the effect of temperature, Chellappan et al. (2005) inoculated cassava seedlings with isolates (Cameroon) of ACMV. Plants expressed severe symptoms 14 days post-inoculation (dpi) at 25 °C and 7 dpi at 30 °C. Symptom severity was greater among seedlings kept at 25 °C compared with those at 30 °C.

Considerable variability in CMD symptoms has been observed among Zambia’s three agro–ecological zones (AZI, AZII, and AZIII), which are defined by rainfall, temperature and soil type. Zambia is tropical; temperatures are moderated by altitude. There are three seasons: a cool dry season (April–August), a hot dry season (August–November), and a hot wet season (November–April). The average temperatures range from a mean monthly minimum of about 10 °C in June and July to a mean monthly maximum of 30 °C in October and November. AZI is hot and low-lying and includes the Zambezi and Luangwa valleys (including parts of Lusaka and Eastern provinces) in the south and east, respectively (Jain 2007). It experiences annual rainfall of less than 800 mm. AZII covers the centre of the country, stretching from the western to the eastern borders and is characterised by average annual rainfall of 800–1000 mm. AZIII is the largest zone, stretching from the northwest to the northeast of the country and experiences annual rainfall of 1000–1400 mm. CMD symptoms are much more pronounced in AZI than in AZII or AZIII, partly as a result of the higher temperatures as shown by surveys in 2009 and 2014 (Chikoti et al. 2013a, 2015). For example, in the 2009 survey, CMD severity on a 1–5 scale (Hahn et al. 1980) for Lusaka and Eastern provinces was reported to be 3.88 and 3.94, respectively (Chikoti et al. 2013a). In the 2014 survey, severity was 3.48 and 3.14 for Lusaka and Eastern provinces, respectively (Chikoti et al. 2015).

Another potential factor in disease expression is the presence of subviral agents called sequence enhancing geminivirus symptoms (SEGS) (formerly satellite DNA molecules). These satellite DNA molecules are known to modulate replication and symptom expression of their helper virus (Maredza et al. 2016; Ndunguru et al. 2016). SEGS associated with cassava begomoviruses have been shown to enhance severe symptoms and break down plant resistance (Ndunguru et al. 2008). In Zambia, CMD infected cassava leaf samples collected from Luapula Province tested positive for SEGS II and III with primer pairs SatIIR/F and SatIIIR/F designed by Ndunguru et al. (2008). Severe leaf symptoms were observed on plants that tested positive for SEGS. These symptoms included crumpling, curling and leaf narrowing.

## Causal organism

At least seven geminiviruses have been reported to cause CMD in Africa (Hillocks and Thresh 2000). These are ACMV (Stanley and Gay 1983), EACMV (Hong et al. 1993), South African cassava mosaic virus (SACMV) (Berrie et al. 2001), East African cassava mosaic Kenya virus (EACMKV) (Bull et al. 2006), East African cassava mosaic Zanzibar virus (EACMZV) (Maruthi et al. 2004), East African cassava mosaic Cameroon virus (EACMCV) (Fondong et al. 2000), and East African cassava mosaic Malawi virus (EACMMV) (Zhou et al. 1998). Among these species, several variants have been described, some of which are products of inter- and intraspecific recombinations. For example, the Ugandan variant of EACMV (EACMV-UG) (Zhou et al. 1997) is a widely reported recombinant (Pita et al. 2001b; Were et al. 2004; Ndunguru et al. 2005) of EACMV and ACMV that developed through interspecific recombination (Zhou et al. 1997).

Advances in molecular techniques have broadened diagnostic capabilities and enabled broader surveys of viral diseases on crops. Mkuyamba (1995) was the first to detect ACMV and cassava Q virus using an indirect triple antibody sandwich (TAS) enzyme-linked immunosorbent assay (ELISA) and an immunosorbent electron microscope (ISEM). Leaf samples collected from Lusaka, Luapula, Northern, North-Western, Central and Copperbelt provinces were tested and viral identity confirmed at the University of Zambia and Scottish Crops Research Institute in the United Kingdom. Later, in 1996, Ogbe et al. (1997) used the biotinylated monoclonal antibodies (mAb) SCR 23 and SCR 33 to detect ACMV and EACMV during a survey of Luapula Province. Following the work of Ogbe et al. (1997), comprehensive surveys were carried out between 2009 and 2015.

In 2014, Mulenga et al. (2015) used polymerase chain reaction (PCR), cloning and sequencing, and reported EACMMV in Zambia. A nucleotide BLAST (BLASTX) search in the National Center for Biotechnology Information (NCBI) GenBank of partial sequences that was obtained from direct sequencing of PCR products (Tembo 2016), showed substantial homology of the Zambian isolates with sequences of ACMV-UGMild Uganda (AF126800.1), ACMV-UGSvr Uganda (AF126802.1), ACMV-[MG:MG310A1] Madagascar and ACMV-CM39 Cameroon (AY211462.1). Sequence identities were between 97% and 98%. Within the EACMV species, the isolates showed greater variability, with sequence divergence between 77% and 99% (Tembo 2016). In another study, core coat protein gene sequences of Zambian isolates (KT869078 to −118) clustered with several isolates in the seven geminivirus species without resolving into specific clades, particularly among the EACMV-like viruses (Mulenga et al. 2016). A comparison of the virus sequences obtained from GenBank with the complete DNA-A genome isolates characterised from selected EACMV-like Zambian isolates (KT869123 to 126) revealed >92% AV1 sequence identity with the AV1 regions of EACMV-CM (AF112354), EACMV-MW (JX473582), EACMV-TZ (AY795983) and EACMV-KE (AJ717542) and 84% to 89% for EACMV-UG (AF126804). However, a comparison of the complete DNA-A genome sequence of the same isolates showed 85% to 88% identity for EACMV-CM, EACMV-MW and EACMV-TZ, 94% for EACMV-KE, and 92% for EACMV-UG (Mulenga et al. 2016). The variation in sequence identity suggests that more viruses remain undetected and underlines the need for further studies.

## Distribution of cassava mosaic begomoviruses and prevalence of CMD

**Fig. 2:**
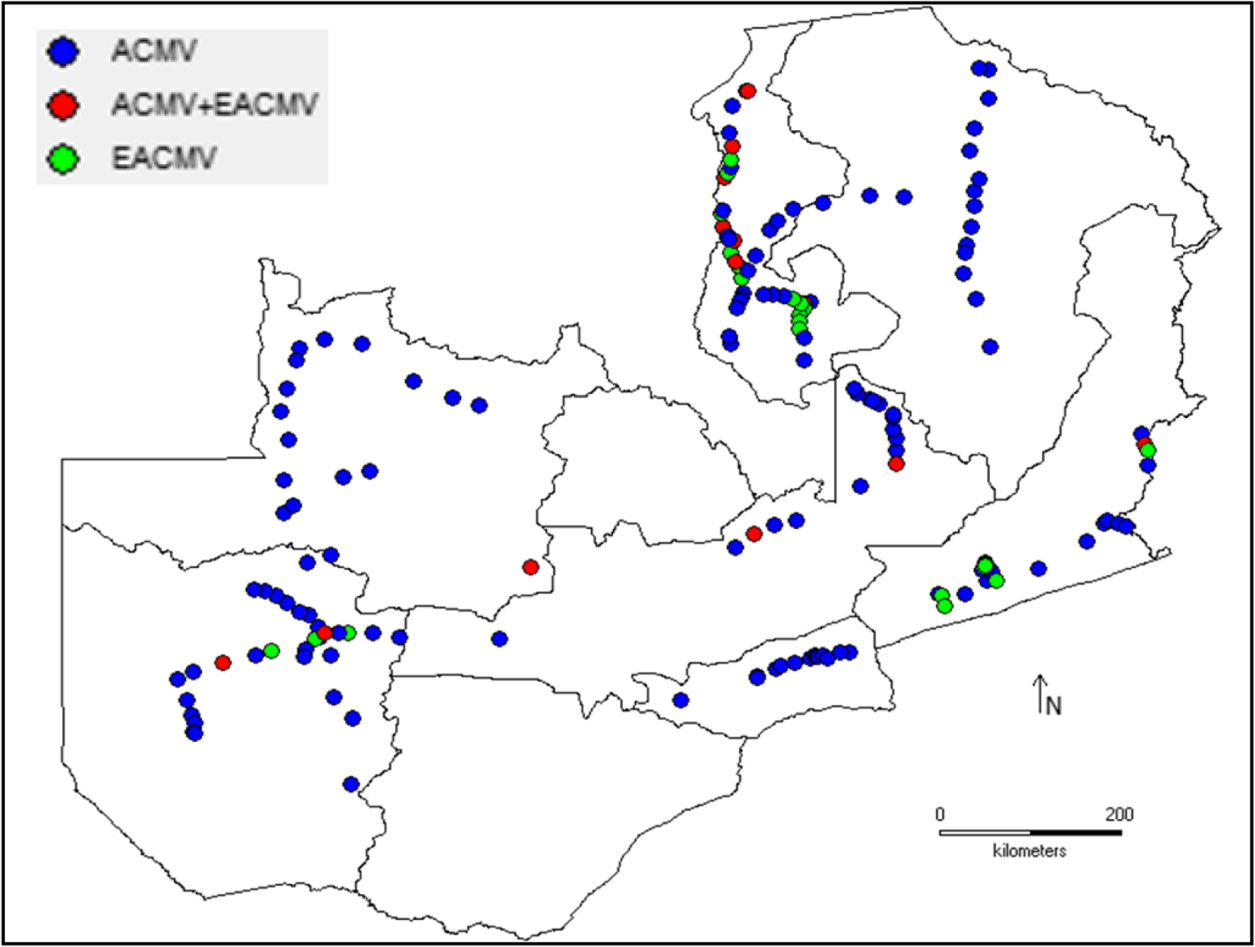
Distribution of cassava mosaic begomoviruses in 2009: African cassava mosaic virus (ACMV) and East African cassava mosaic virus (EACMV)

CMD is widely distributed in Zambia. Among the viruses causing CMD, reports have shown that ACMV is more widespread than EACMV-like viruses. Mkuyamba (1995) detected only ACMV in the samples collected from 20 locations in six provinces (Lusaka, Luapula, Northern, North-Western, Central and Copperbelt) using mAb that included SCR23 and SCR33 targeted against ACMV. Although SCR33 reacts only with ACMV, SCR23 can react with both ACMV and EACMV. Ogbe et al. (1997) exploited this capability to screen 100 samples from Luapula Province for the presence of both ACMV and EACMV. ACMV and EACMV was detected in 81% and 6% of the samples, respectively. In a more thorough survey conducted in the 2009–2010 growing season, Chikoti et al. (2013a) used PCR assays with pairs of genus-specific oligonucleotides (JSP01/02 and EAB555F/R, Pita et al. 2001b; Fondong et al. 2000) to discriminate between ACMV and EACMV in samples that were collected from seven provinces in Zambia. They found ACMV in 65.4% of samples tested and EACMV in 25% of them. They also reported the first dual infection of ACMV and EACMV in Zambia, which occurred in 9.6% of the samples (Fig. 2; Chikoti et al. 2013a). Subsequent surveys have found generally similar estimates and widespread distributions for both viruses. In 2014, Chikoti et al. (2015) reported ACMV, EACMV, and dual infection of ACMV and EACMV in 67.9%, 6.8% and 25.6% of the samples, respectively. Recently, Mulenga et al. (2016) also found that EACMMV was widespread, with samples collected from six of the seven provinces surveyed.

The high prevalence of CMD in Zambia is largely attributed to the continuous cultivation of susceptible cassava cultivars and the indiscriminate use of diseased planting materials (Chikoti 2011; Tembo 2016). In 1996, CMD incidence was first reported to be 40.8% in Zambia (Muimba-Kankolongo et al. 1997). By 2009, CMD incidence was 52% (Chikoti et al. 2013a). In Lusaka and North-Western provinces, the incidence of CMD was 67.4% and 71.2%, respectively in 2009 (Chikoti et al. 2013a). In Rufunsa district, where yields have been poor and disease incidence as high as 100% has been recorded, some farmers have abandoned their fields or opted to grow crops other than cassava (Tembo, personal observation).

## Transmission and epidemiology

The use of infected planting materials is one of the main causes for the spread of CMD. In Zambia, like in many other African countries, cassava is usually propagated using hardwood stem cuttings. Most subsistence farmers either recycle cassava planting materials or obtain them from their friends, neighbours, middlemen, non-governmental organisations (NGOs) or Agricultural Research Stations (Simwambana 2005). There is no formal cassava seed system in Zambia, so the materials sold by seed multipliers and shared among farmers are not properly checked or certified as clean planting material. As a result, most of the cassava planting materials sold and shared in Zambia are diseased.

**Fig. 3:**
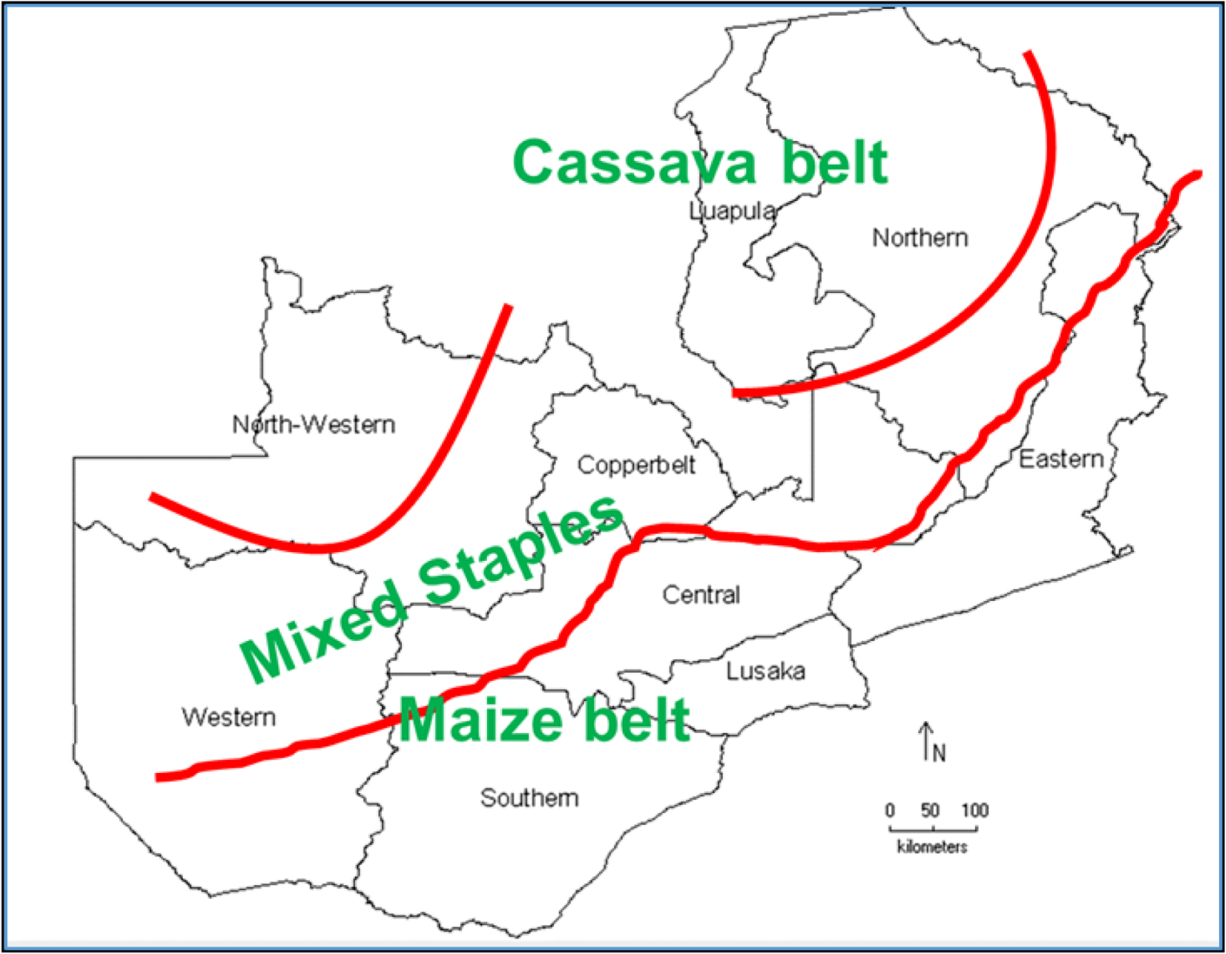
Growing zones of common crops in Zambia

Cassava mosaic geminiviruses are principally transmitted by whitefly (order Hemiptera, family *Aleyrodidae*), which move the virus from plant to plant within a field as well as among nearby fields. Although there has been considerable research on the transmission of CMD viruses by *B. tabaci* in Uganda, Tanzania and Kenya (Legg et al. 2002; Murgerwa et al. 2012), similar research has not been done in Zambia. A few surveys have shown relatively high whitefly populations in parts of Western and Lusaka provinces, particularly in Kaoma and Rufunsa districts, respectively (Chikoti et al. 2013a, 2015; Tembo 2016). In some fields, more than 100 whiteflies have been recorded per plant (Chikoti et al. 2013b). During the Uganda CMD pandemic in the 1990s, the rapid spread (10–20 km/year) of the disease (Gibson et al. 1996; Otim–Nape et al. 2000) was fuelled by unusually high whitefly populations on the CMD-resistant cultivars that were introduced from the International Institute of Tropical Agriculture (IITA) (Legg and Ogwal 1998). In the cassava belt (Fig. 3), Northern and Luapula provinces, whitefly populations are low (Chikoti et al. 2013a); consequently, spread of CMD is mostly through the use of diseased planting material.

## Effect on crop growth and yield

**Fig. 4:**
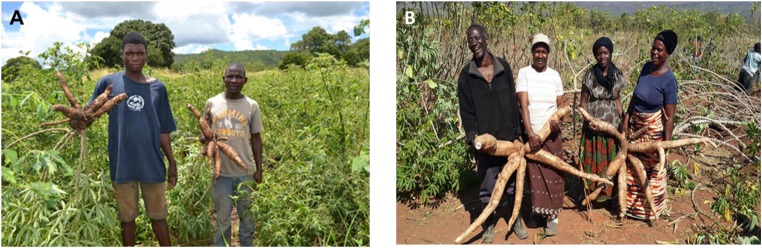
**a** Cassava plants infected with cassava mosaic disease (CMD) exhibiting reduced tuber size. **b** Healthy cassava plants with relatively large tubers

In other parts of Africa, there are extensive data on how CMD impacts plant growth and cassava yields (Fargette et al. 1988; Fauquet and Fargette 1990; Owor et al. 2004; Thresh and Cooter 2005). In Zambia, available data from surveys and field assessments are limited, but the data that has been collected from farmers’ fields and experimental research plots in Zambia, generally show a reduction in plant growth when CMD is present (Tembo 2016). CMD causes chlorosis in cassava leaves, which reduces photosynthetic activity and ultimately leads to reduced plant growth, lowered yields, or the complete loss of that season’s yield (Fig. 4).

Although cassava yields are difficult to measure in farmers’ fields as a result of irregular and partial harvests, Muimba–Kankolongo et al. (1997) estimated that the reduction in crop yields in Zambia as a result of CMD was between 50% and 70%. In a more recent report (Tembo 2016), data collected from field experiments indicated even higher yield losses. Clearly, the consequences of CMD are devastating and the disease is one of the major constraints to maintaining sufficient crop yields. Thresh et al. (1997) estimated yield losses in Africa to be 15–28 million tonnes representing 15% to 24% of total cassava production. The estimated annual economic losses in East and Central Africa are between $1.9 and $2.7 billion USD (Patil and Fauquet 2009).

## Range of plant hosts

Cassava-infecting geminiviruses cause the most economic damage in cassava. The range of plant hosts that a particular virus can infect depends on the species of virus. Viral species that can transmit and cause CMD on cassava also infect plants in the genera *Nicotiana* and *Datura* (Bock and Woods 1983). Viruses that cause CMD on cassava have also long been reported to infect the closely related euphorbiaceous species, *Jatropha multifida* (Bock et al. 1978). Although *J. multifida* is found in Zambia, CMD symptoms have not yet been observed on this plant species. Work to identify non-cassava plants that could be alternative hosts of cassava mosaic begomoviruses (CMBs) in Zambia is ongoing. In West Africa, the information available suggests CMBs occur on non-cassava plants, including *Senna occidentalis* (L.), *Leucana leucocephala* (Lam.), *Combretum confertum* (Benth.) and *Manihot glaziovii* (Alabi et al. 2008).

## Emerging threats to cassava production in Zambia

Cassava brown streak disease (CBSD) is a threat to Zambia. CBSD is quite devastating; it affects all plant parts and more importantly it causes the roots to rot, thus making them unfit for human consumption (Nicholas 1950). In coastal East Africa and around the Great Lakes region, CBSD is considered the biggest threat to food security (Mohammed et al. 2012). In Zambia, the disease was first diagnosed in North Western Province during the routine disease diagnostic surveys in the first quarter of 2017 (Tembo et al. 2017). Subsequent surveys in the second quarter of 2017 in Luapula and Northern provinces showed 32.3% CBSD incidence (Mulenga et al. 2018).

## Management strategies

The importance of CMD lies in the fact that it is widespread in Zambia and causes huge yield losses. Several approaches have been used or proposed to manage CMD, but the two conventional ones are planting CMD-resistant cultivars and implementing phytosanitary protocols. The development of CMD-resistant plants started soon after it was recognised that CMD was caused by a virus (Storey and Nichols 1938) and continued thereafter (Nichols 1947; Jennings 1957; Hahn et al. 1980). Efforts from these early studies fed into the IITA breeding programme and resulted in the release of CMD-resistant tropical *Manihot* selections (Jennings 1994; Beck 1982; Nweke et al. 2002). These selections provided sources of resistance genes, and have been used in breeding programmes in many countries, including Zambia. Phytosanitation has also been cited extensively as an important component of CMD management strategies (Legg 1999; Legg and Fauquet 2004; Thresh and Cooter 2005). The main strategies to manage CMD in Zambia are: (i) improved diagnostics and monitoring, (ii) phytosanitation, (iii) distribution of disease-free planting material, (iv) use of CMD-resistant cassava cultivars, (v) training and outreach, and (vi) stakeholder networks.

### Diagnostics and monitoring

To foster a comprehensive approach to solving the CMD problem, a diagnostic laboratory has been strengthened at the ZARI–Mt. Makulu Research Station with the support of the project,” Disease Diagnostics for Sustainable Cassava Productivity in Africa,” which is based in Tanzania, but is implemented in six other countries: Malawi, Mozambique, Kenya, Rwanda, Uganda and Zambia. Three countrywide surveys were conducted in 2009, 2013 and 2015 to monitor and assess the status of CMD in the major cassava-producing provinces in Zambia. Data from these surveys have been used to develop prevalence maps indicating the detailed distribution of CMBs, CMD incidence and severity, and whitefly populations (Chikoti and Matimelo 2014). In addition, these maps have been used to inform policy makers, agricultural research and educational institutions, (such as universities and colleges), extension agents and NGOs that are involved in Zambia’s cassava subsector.

### Phytosanitation

Although phytosanitation is a key component in the management of CMD, it has rarely been applied in the field in Zambia. The key feature of phytosanitation is crop hygiene, with an emphasis on using disease-free planting materials, and removing diseased plants. Crop hygiene also involves the removal of debris and surviving plants from the previous crops to reduce carrying diseased inoculum to new fields (Fargette et al. 1990). Although this approach has been used in other countries, in Zambia, most farmers do not remove diseased materials, largely because they lack knowledge of the disease and its management. Phytosanitary procedures have great potential to reduce the disease, specifically when the provision of clean planting materials follows massive removal of infected field stock. For example in western Kenya where selection was applied, plots of CMD-susceptible cultivars gave tuberous yield that exceeded those of the control and were comparable to the resistant cultivar grown under similar conditions (Mallowa et al. 2014).

### Disease-free planting material

The major source of cassava planting material is from a farmer’s own fields, from neighbours (Chikoti 2011), and sometimes from cassava marketing middlemen. Although farmers plant healthy-looking cassava stems, most of these stems are infected with CMD. Thus, managing CMD at the planting stage is ineffective. Instead, development and provision of disease-free planting materials are recognised as superior control strategies to manage CMD (See Table 1 for a summary of research activities related to diseases affecting cassava production in Zambia). In Tanzania, success in increasing cassava yields has been achieved through the use of disease-free planting materials (IITA 2017). In 2013, to advocate for the use of virus-free materials, ZARI, in collaboration with cooperating partners, established a tissue-culture laboratory to produce disease-free planting materials. This laboratory is still operational, but it lacks adequate staff and reagents.

**Table 1:** Summary of relevant events related to cassava production and cassava mosaic disease in Zambia between 1993 and 2014

Event	Year	Province	Reference
Release of the first high-yield local cultivars	1993	Countrywide	Haggblade and Nyembe 2008
First report of ACMV and cassava Q virus	1995	Luapula, Northern, North-Western, Central, Copperbelt, Lusaka*	Mkuyamba 1995
First countrywide survey of CMD; incidence reported to be 40.8%	1995 and 1996	North-Western, Luapula, Northern, Central, Western, Copperbelt	Muimba–Kankolongo et al. 1997
Release of the first CMD-resistant cultivars	2000	Countrywide	Haggblade and Nyembe 2008
CMD incidence reported to be 46%	2006	Northern, Luapula	J.P Legg and P.C Chikoti 2006
Comprehensive countrywide CMD survey; disease incidence reported to be 52%	2009	Eastern, Western, Lusaka, Central, Northern, Luapula, North-Western	Chikoti et al. 2013a
First report of EACMMV in Zambia	2014	Western, Northern, Luapula, Lusaka, Eastern	Mulenga et al. 2016

*ACMV* African cassava mosaic virus, *CMD* cassava mosaic disease, *SACMV* South African cassava mosaic virus, *EACMMV* East African cassava mosaic Malawi virus. *Tested for cassava Q virus and ACMV

Research conducted at Mansa Research Station and in Lusaka Province from 2002 to 2004 demonstrated the marked effect of using CMD-free local cassava cultivars as well as using improved cultivars with respective yield gains of 21% and 41% over the control cultivar (Munganga) (Alene et al. 2013). Although there are about 59 cassava seed multipliers in the country (SCCI 2016), these multipliers do not test planting materials for viruses. It was not until 2013 that the Seed Control and Certification Institute (SCCI), a government seed regulatory agency, put in place certification standards for vegetative propagated planting materials (PVSR 2016).

### Use of CMD-resistant cultivars

The Root and Tuber Improvement Programme (RTIP), which falls within ZARI, has developed and released cassava cultivars that outperform the local cultivars by using breeding materials from the IITA. Initially, RTIP focused on the identification of the best local cultivars and on the cleaning and distribution of planting materials. In 1993, it released three cultivars, Bangweulu, Kapumba and Nalumino (Table 2; Haggblade and Nyembe 2008), which have higher yields (22–31 t/ha). However, despite being considered disease resistant (Soenarjo 1992), these cultivars are susceptible to CMD (Tembo 2016). In 2000, following the release of the best-performing local cultivars (Bangweulu, Kapumba and Nalumino), four high-yield cultivars, Chila, Kampolombo, Mweru and Tanganyika, which are resistant to CMD were released. To support cassava breeding programmes, the project,” Disease Diagnostics for Sustainable Cassava Productivity in Africa,” is focused on screening and indexing germplasm and parental materials for resistance breeding. However, getting farmers to use these improved and resistant cultivars remains a challenge. Local cassava cultivars continue to dominate; it is estimated that local cultivars are planted on over 70% of all cassava-growing areas (Alene et al. 2013). Awareness and access to planting materials are important factors in the adoption of improved cultivars. Equally, farmer participation in the development of new cultivars is necessary. Adoption will not take place unless farmers are aware of which cultivars exist and what their attributes are before getting access to the planting materials.

**Table 2:** Improved cassava cultivars released in Zambia between 1990 and 2000

Cultivar	Category	IITA material	Year of release	Yield (t/ha)	Maturity (MAP)	Taste
Bangweulu	Local selection	None	1993	31	12–16	Bitter
Kapumba	Local selection	None	1993	22	16–24	Sweet
Nalumino	Local selection	None	1993	29	16–24	Bitter
Mweru	Improved	IITA male x Nalumino	2000	41	16	Sweet
Chila	Improved	IITA male x Nalumino	2000	35	16	Bitter
Tanganyika	Improved	IITA male x Nalumino	2000	36	16	Sweet
Kampolombo	Improved	IITA male x Nalumino	2000	39	16	Sweet

Source: Haggblade and Nyembe (2008)

MAP, months after planting

### Training and outreach

Although CMD has been observed in fields for decades, most farmers are unaware of the disease or its impact. In a study carried out between 1995 and 1996 in Northern, North-Western, Central, Luapula and Western provinces, only one farmer out of 121 was familiar with CMD (Muimba–Kankolongo et al. 1997). More recently, in 2009, a survey conducted in Luapula Province showed only three out of the 120 farmers sampled knew CMD (Chikoti 2011). Farmers do not usually attempt to control CMD because their knowledge of how to recognise and manage it is limited and requires specialised training. A key component of the project “Disease Diagnostics for Sustainable Cassava Productivity in Africa” is training extension personnel to recognise and manage the disease. More than 1000 farmers have now been trained at field days and demonstration plots that have been established in Kaoma, Mansa and Rufunsa districts.

In addition to training farmers, long- and short-term training has been provided to ZARI staff and has resulted in the development of diagnostic tools for virus identification in Zambia (Chikoti 2010). Various stakeholders from the University of Zambia, SCCI, Plant Quarantine and Phytosanitary Service, and extension personnel have also been trained in various diagnostic techniques, including symptom recognition and management. Furthermore, information on the importance of the disease, its spread and its impact have been disseminated through radio and television documentaries, national and provincial agricultural shows, brochures and leaflets. These efforts should continue to be strengthened to reach a wider audience.

### Stakeholder networks

The key to successfully reducing the impact of CMD depends on the active participation of key players in the cassava sector. These include scientists, extension workers, seed multipliers, seed certifiers, NGOs, and policy makers. Although research and extension have existed for more than 40 years within the Ministry of Agriculture, communication flow between these institutions remain weak. Information generated through research is not easy for farmers to access. As the communication flow improve, small-scale farmers will benefit in the long term as a result of accessing the correct information at the right time, including strategies for managing CMD.

## Conclusion

CMD continues to be a major threat to cassava production in Zambia. Infection induces several morphological and cytological alterations, often resulting in significant losses in yield that ultimately reduce farmers’ income. Despite the development and release of improved cassava cultivars, the adoption of these cultivars by farmers remains a challenge that is illustrated by the increase in CMD incidence between 1997 and 2009, as a result of a continued use of susceptible local cultivars. Although there is now renewed interest by the Ministry of Agriculture in tackling the CMD scourge, much work still needs to be done to continue raising awareness of the disease among policy makers, farmers and extension agents. We suggest that future management efforts address the following:

- Given farmers’ preference for local cassava cultivars, focus attention and resources on cleaning planting materials using tissue-culture-based techniques.
- Consider farmers’ perceptions of the attributes of local cultivars. To improve adoption of new cultivars, cassava breeding programmes should involve farmers during the earliest selection stages of the development of new high-yielding and pest-resistant cultivars.
- Train farmers in phytosanitary practices.
- Improve and strengthen the cassava seed production and distribution system.
